# Analysis of mitotic phosphorylation of Borealin

**DOI:** 10.1186/1471-2121-8-5

**Published:** 2007-01-22

**Authors:** Harpreet Kaur, Andrew C Stiff, Dipali A Date, William R Taylor

**Affiliations:** 1 Department of Biological Sciences, University of Toledo, 2801 W. Bancroft Street, MS 601, Toledo, OH 43606, USA

## Abstract

**Background:**

The main role of the chromosomal passenger complex is to ensure that Aurora B kinase is properly localized and activated before and during mitosis. Borealin, a member of the chromosomal passenger complex, shows increased expression during G2/M phases and is involved in targeting the complex to the centromere and the spindle midzone, where it ensures proper chromosome segregation and cytokinesis. Borealin has a consensus CDK1 phosphorylation site, threonine 106 and can be phosphorylated by Aurora B Kinase at serine 165 *in vitro*.

**Results:**

Here, we show that Borealin is phosphorylated during mitosis in human cells. Dephosphorylation of Borealin occurs as cells exit mitosis. The phosphorylated form of Borealin is found in an INCENP-containing complex in mitosis. INCENP-containing complexes from cells in S phase are enriched in the phosphorylated form suggesting that phosphorylation may encourage entry of Borealin into the chromosomal passenger complex. Although Aurora B Kinase is found in complexes that contain Borealin, it is not required for the mitotic phosphorylation of Borealin. Mutation of T106 or S165 of Borealin to alanine does not alter the electrophoretic mobility shift of Borealin. Experiments with cyclohexamide and the phosphatase inhibitor sodium fluoride suggest that Borealin is phosphorylated by a protein kinase that can be active in interphase and mitosis and that the phosphorylation may be regulated by a short-lived phosphatase that is active in interphase but not mitosis.

**Conclusion:**

Borealin is phosphorylated during mitosis. Neither residue S165, T106 nor phosphorylation of Borealin by Aurora B Kinase is required to generate the mitotic, shifted form of Borealin. Suppression of phosphorylation during interphase is ensured by a labile protein, possibly a cell cycle regulated phosphatase.

## Background

The chromosomal passenger complex (CPC) consisting of Aurora B kinase, INCENP (INner CENtromere Protein), Survivin and Borealin/Dasra B plays important roles during mitosis and cytokinesis [1]. One of the main aims of the CPC proteins is to ensure that Aurora B is accessible to phosphorylate its various substrates like histone H3, CENP-A, MKLP1, MCAK, INCENP, Survivin, MgcRacGAP, Vimentin, Desmin and myosin-II [2-14] at the right time. Thus, the CPC proteins regulate multiple mitotic events like chromosome segregation, operation of the spindle assembly checkpoint and cytokinesis [1]. How phosphorylation by Aurora B affects the functions of its various substrates and hence influences cell division is not completely understood.

The CPC proteins concentrate at the inner-centromere during metaphase, migrate to the spindle midzone during anaphase and finally to midbody during cytokinesis [1]. The exact mechanism of this characteristic localization of the CPC is currently unknown, however clues are emerging. Borealin and Survivin can self-associate *in vitro *and *in vivo *and can also interact with each other [15-20]. Borealin can bind to DNA *in vitro *[20]. Also, the BIR-domain of Survivin has been proposed to interact with the centromeres [21]. Furthermore, a complex of Borealin, Survivin and the N-terminus of INCENP (1–58) is capable of targeting to the centromere *in vivo *[20]. Borealin and Survivin may act as a scaffold to bring INCENP and Aurora B Kinase to the centromere. INCENP can bind to tubulin directly thereby targeting the CPC to the spindle midzone [22-24].

Several members of the CPC are regulated by post-translational modification. For example, INCENP is phosphorylated by Aurora B and CDK1, both of which enhance the ability of INCENP to activate Aurora B [25,26]. Also, the phosphorylation of INCENP by CDK1 allows it to interact with Plk1 and recruit it to the centromere [26]. There is also evidence that Survivin is regulated by phosphorylation [10]. Borealin co-localizes with Aurora B Kinase and can be phosphorylated at serine 165 by Aurora B Kinase *in vitro *[18]. Here, we show that Borealin is phosphorylated *in vivo *during mitosis as indicated by an electrophoretic mobility shift. Aurora B is not required for this particular modification. Mutation of S165, potential Aurora B Kinase phosphorylation site, to alanine did not alter the mitotic phosphorylation of Borealin or its localization to the centromere, spindle midzone or midbody indicating that other sites are targets of modification *in vivo*.

## Results

### Two electrophoretic forms of Borealin in human cells

**Figure 1:**
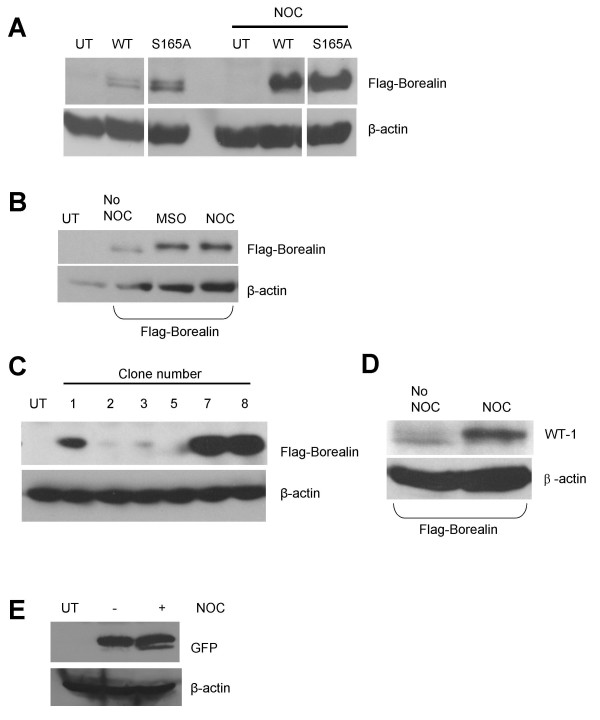
Mobility shift of exogenous Borealin. Mobility shift and protein levels of exogenous Borealin in (A), (B), (C) and (D) were determined by Western blotting using an antibody to the Flag-tag. Transfers were stripped and reprobed for β-actin as a loading control. UT: untransfected. The multiple blocks in (A) are bands from the same gel. (A) Two electrophoretic forms of Borealin. Asynchronously growing Hela cells were transiently transfected with either wild type or S165A phosphomutant of Borealin. Transfected cells were either left untreated or treated with nocodazole for 14 hours to block cells in mitosis. Cell lysates were separated using 15% SDS-polyacrylamide gel. (B) Mobility shift of exogenous Borealin. Mitotic WT-8 cells were collected either by mitotic shake-off or by using nocodazole. Cell lysates were separated on a 12.6% SDS-polyacrylamide gel and analyzed by Western blotting. Lysates were less concentrated than those used in "A" and in asynchronous cells (No NOC) only the more abundant faster migrating form is visible. (C) Expression level of ectopic Borealin in different clones. Clones of Hela cells stably expressing different levels of wild-type Flag-Borealin were analyzed by Western blotting using 12.5% SDS-polyacrylamide gel. (D) Mobility shift of Borealin in WT-1. WT-1 cells stably expressing a lower level of wild-type Flag-Borealin than WT-8 were either left to grow asynchronously (No NOC) or blocked in mitosis using nocodazole (NOC). Cell lysates were separated on a 12.6% SDS-polyacrylamide gel. (E) Expression of GFP in Hela cells. Hela cells were transiently transfected with CMV-*gfp *and left to grow either asynchronously or treated with nocodazole. Cell lysates were separated by 12.5% SDS-polyacrylamide gel and subjected to Western blotting with anti-GFP antibody.

During our analysis of the expression of Flag-tagged Borealin protein, we periodically observed two bands. Therefore, we transiently transfected Hela cells with WT Flag-Borealin and separated the extracts by more extensive electrophoresis using a modified acrylamide/bisacrylamide ratio (see Methods). Under these conditions, we found that Borealin could be resolved into a doublet (Fig. 1A, compare UT to WT). The presence of two migrating forms suggests that Borealin may be post-translationally modified in cells. Furthermore, we observed that cells blocked in mitosis with nocodazole contained mostly the slowly migrating form whereas asynchronously growing cells contained the faster form (Fig. 1B). Nocodazole arrests cells in mitosis by preventing microtubule polymerization which activates the spindle checkpoint. This raised two possibilities, either that Borealin was modified as cells naturally entered mitosis, or that it was modified as a consequence of triggering the spindle checkpoint.

Mitotic WT-8 cells collected by mitotic shake-off in the absence of nocodazole contained mostly the slowly migrating form of Borealin, similarly to nocodazole blocked cells (Fig. 1B). This suggests that the modification of Borealin that we have identified occurs as cells naturally enter mitosis, and is not a consequence of activation of the spindle checkpoint. In addition, the mitosis-specific mobility shift of wild-type Borealin was observed in two independent stable clones that express different levels of the ectopic protein (Fig. 1C and 1D). This suggests that phosphorylation is not clone specific and can be seen when lower levels of the protein are expressed.

**Figure 2:**
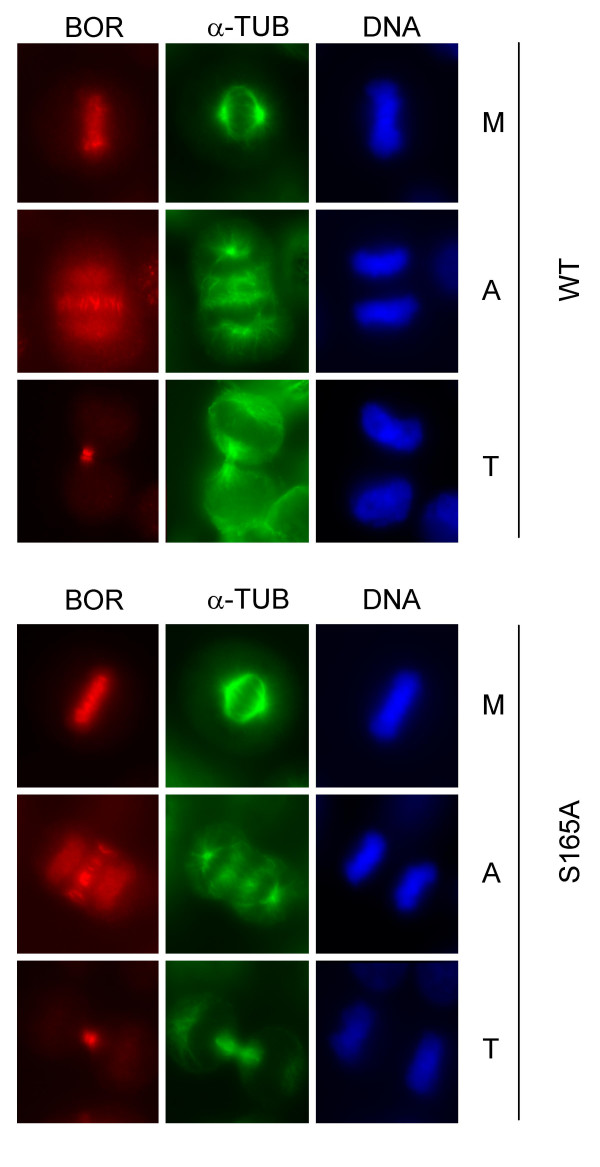
Localization of ectopic wild type or S165A mutated version of Borealin. Hela cells were transiently transfected with wild type or S165A phosphomutant of Borealin. Localization of ectopic Borealin (BOR-red), α-tubulin (α-TUB-green) and DNA (DNA-blue) in cells was analyzed using fluorescence microscopy. M denotes Metaphase, A denotes Anaphase and T denotes Telophase.

Borealin is found in a complex with Aurora B, a serine/threonine kinase that is active during mitosis and not interphase [18,19]. Also, Aurora B can phosphorylate serine 165 of Borealin *in vitro *[18]. One possibility was that the mobility shift we observed during mitosis was due to phosphorylation of the protein by Aurora B Kinase. Therefore, we mutated S164 and S165 to alanine (hereafter named S165A) and transiently transfected the mutant into Hela cells. Borealin migrated as a doublet even when S165A was mutated to alanine (Fig. 1A) suggesting that phosphorylation of S165 is not required to generate the shifted form of Borealin. Also, the S165A form of Borealin localized to the centromeres during metaphase, to the spindle midzone during anaphase and to the midbody during telophase similarly to wild-type Borealin (Fig. 2). By database searching, we found that T106 of Borealin conforms to the consensus for CDK1 phosphorylation. Similarly to S164/5, mutation of T106 to alanine did not reduce the mobility shift of the protein in mitosis and had no effect on its subcellular localization (our unpublished data).

### Analysis of endogenous Borealin

**Figure 3:**
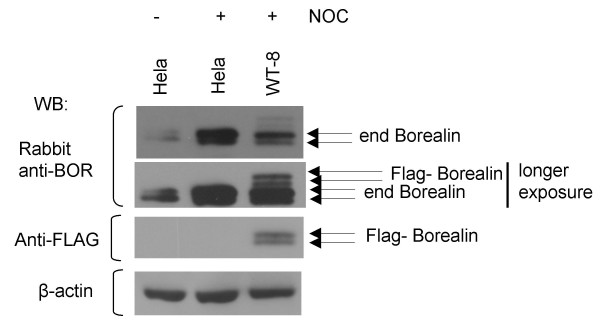
Mobility shift of endogenous Borealin. Cell lysates of asynchronously growing and mitotic Hela and WT-8 cells were separated using 12.6% SDS-polyacrylamide gel and assessed by Western blotting (WB) using antibodies to endogenous (end) Borealin or the Flag-tag. Transfers were stripped and reprobed for β-actin as a loading control.

To analyze endogenous Borealin, we raised an antiserum to the human protein. Asynchronously growing and mitotic Hela cells were assessed by Western blotting to determine if endogenous Borealin showed a mobility shift. Western blot analysis of untransfected, Hela cells using the antibody to Borealin revealed the presence of two electrophoretic forms of Borealin (Fig. 3). Also, mitotic cells showed an increase in the slowly migrating form of Borealin similar to the Flag-tagged Borealin. Western blot analysis of WT-8 cells containing the Flag-tagged Borealin using our antibody to Borealin revealed four bands during mitosis, with the upper two bands being recognized by the antibody to the Flag-tag (Fig. 3). The slower migration of the Flag-tagged Borealin is apparently due to the extra 8 amino acids comprising the tag. Also, the Flag-tagged Borealin in WT-8 cells appears to be less abundant than the endogenous protein. These results indicate that our observations with the Flag-tagged protein are not due to overexpression.

### Phosphorylation of Borealin during mitosis

**Figure 4:**
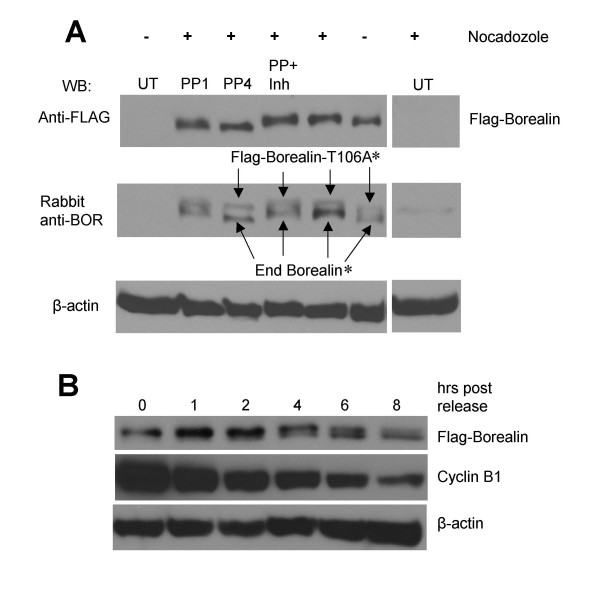
Phosphorylation of Borealin in mitosis. Hela cells were transfected with Flag-Borealin that was analyzed by Western blotting with an antibody to the Flag-tag and endogenous Borealin. (A) Mitotic phosphorylation of Borealin. Hela cells transiently transfected with the T106A mutant of Borealin were either allowed to grow asynchronously or blocked in mitosis using nocodazole. Lysates from transfected, mitotic cells were treated with phosphatase for 1 hrs (PP1) and 4 hrs (PP4) *in vitro *and also with phosphatase inhibitor (PP+Inh). Cell lysates were run on a 15% or 12.6% SDS-polyacrylamide gel. * : Nocodazole treated cells, only the upper, shifted form of Flag-tagged and endogenous Borealin is observed. (B) WT-8 cells were blocked in mitosis using nocodazole and then released into fresh medium. Cells were harvested at the indicated time points and cell lysates were separated by a long run on a 12.6% SDS-polyacrylamide gel. Membranes were stripped and reprobed for Cyclin B1 as a marker for progression through the cell cycle and for β-actin as a loading control.

To determine if the electrophoretic mobility shift of Borealin is due to phosphorylation, mitotic extracts of Hela cells transiently transfected with Flag- Borealin were treated with phosphatase for one and four hours and as a control, with phosphatase and phosphatase inhibitor. The disappearance of the slower migrating form of Borealin upon phosphatase treatment suggests that the slower mobility is due to phosphorylation (Fig. 4A). The fact that adding phosphatase inhibitor blocked the ability of the phosphatase to convert the slower migrating band to the faster migrating band further confirms that the protein is phosphorylated, and that the conversion between forms is not due to contamination of the phosphatase with other enzymatic activities (Fig. 4A). These results demonstrate that Borealin is phosphorylated *in vivo *during mitosis. In the experiment shown, a clone stably expressing a phosphomutant of Borealin T106A was analyzed, however similar results were obtained with wild-type Flag-Borealin and endogenous Borealin (Fig. 4A).

The majority of Borealin is dephosphorylated in asynchronously growing cells, and phosphorylated during mitosis. To determine if Borealin is dephosphorylated as cells exit mitosis we synchronized WT-8 cells in mitosis by exposure to nocodazole. Borealin was analyzed by Western blotting at various time points after release from the nocodazole block. Cells were harvested up to 8 hours post release to determine the mobility shift of Borealin and the level of Cyclin B1 as a control for mitotic exit. At 1-hour post release, Borealin is mostly in the slower migrating phosphorylated form (Fig. 4B). At 1-hour post release we consistently observe a ~30% increase in the total level of Borealin protein compared to cells blocked with nocodazole (Fig. 4B). Dephosphorylation of Borealin is just visible at 2 hours and by 8 hours, cells show an abundant, faster migrating, dephosphorylated form of Borealin. Cyclin B1 levels decreased by 8 hours indicating that the cells have exited mitosis (Fig. 4B). Thus, the dephosphorylation of Borealin correlates with Cyclin B1 degradation.

### Levels of ectopic Borealin in mitosis

We observed a higher level of Flag-Borealin protein when cells were arrested in mitosis (Fig. 1A). In addition, the mutant of Borealin in which S165 was changed to alanine also accumulated to higher levels when cells were blocked in mitosis compared to when they were growing asynchronously (Fig. 1A). The Flag-Borealin used is under the transcriptional control of the cytomegalovirus (CMV) promoter. CMV has been reported to be active throughout the cell cycle [27]. To confirm this in our cells we transiently transfected them with green fluorescent protein (GFP) under the control of the CMV promoter. Similar levels of GFP protein were observed in interphase and mitotic cells suggesting that the overexpression of Borealin is not due to changes in transcription (Fig. 1E).

### Mitotic phosphorylation of Borealin is not dependent on Aurora B Kinase

**Figure 5:**
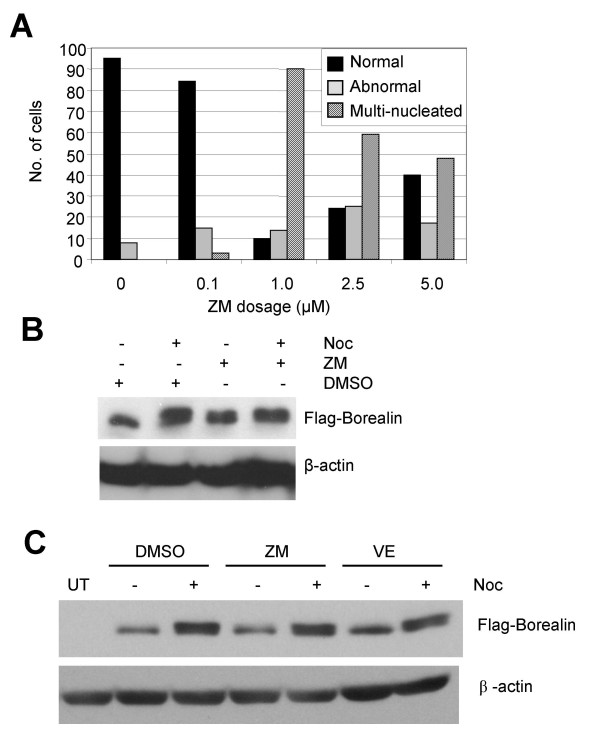
Effect of Aurora kinase inhibitors on the mitotic phosphorylation of Borealin. The mobility shift of Borealin was determined by Western blotting using a 12.6% SDS-polyacrylamide gel. Transfers were stripped and reprobed for β-actin to serve as loading control. (A) The response of cells to different doses of ZM447439. Hela cells were treated for 16 hours with the indicated doses of ZM447439 to block Aurora B kinase activity and then stained to detect DNA using immunofluorescence. The percentage of normal, abnormal and multinucleated cells was determined using fluorescence microscopy. (B) Effect of ZM447439 pre-treatment on mitotic phosphorylation of Borealin. WT-8 cells were treated with ZM447439 for 1 hour and then blocked in mitosis by treating for 16 hours with nocodazole (ZM447439 was left on during the nocodazole treatment). DMSO was used as a vehicle control. (C) Effect of high dose of ZM447439 and VE465 on mitotic phosphorylation of Borealin. WT-8 cells treated with 10 μM ZM447439 or 100 nM VE465 for 1 hour were either left to grow asynchronously or blocked in mitosis by treating for 16 hours with nocodazole (aurora kinase inhibitors were left on for the duration of the experiment). DMSO was included as a vehicle control.

During mitosis, Borealin co-localizes with Aurora B, a mitotic kinase that can phosphorylate S165 of Borealin *in vitro *[18]. Phosphorylation of S165 is not required to generate the mitotic mobility shift of Borealin. However, it was possible that Aurora B phosphorylates a different site to induce the mobility shift. Therefore, we tested the effect of ZM447439, an effective inhibitor of Aurora B kinase activity on the mitotic phosphorylation of Borealin [28,29]. Hela, A549 and HME cells treated with 2 μM ZM447439 enter and exit mitosis but do not undergo cytokinesis [28]. We observed that many Hela cells treated with ZM447439 contained multiple and abnormally shaped nuclei consistent with the inhibition of Aurora B kinase in our treated cells (Fig. 5A). We currently do not have an explanation for the reduction in the percentage of cells at higher doses (5 μM).

To determine the effect of Aurora B Kinase on the phosphorylation of Borealin, we pretreated WT-8 cells with 2 μM ZM447439 for 1 hour and then added nocodazole for 16 hours to block them in mitosis. ZM447439 was left in the medium during the nocodazole treatment to ensure that cells entered mitosis with low levels of Aurora kinase activity. Under these conditions, Borealin migrated as a doublet similar to the cells blocked in mitosis with nocodazole without adding ZM447439 (Fig. 5B). Also, pretreatment of WT-8 cells with a higher dose of ZM447439 (10 μM) or VE465 (100 nM), another Aurora Kinase inhibitor [30] followed by nocodazole did not block the mitotic mobility shift of Borealin (Fig. 5C). This suggests that Aurora B is not required to generate the phosphorylated mitotic form of Borealin.

### Localization of Borealin in cells that fail to divide

**Figure 6:**
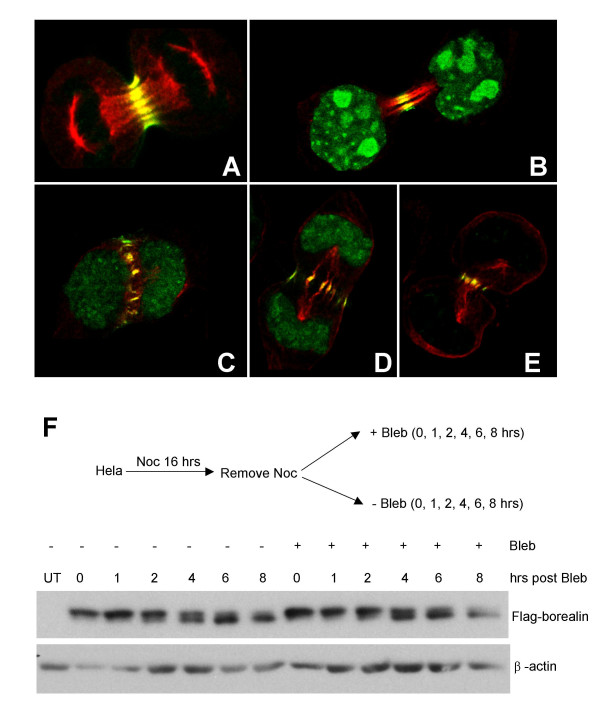
Localization and kinetics of dephosphorylation of Borealin in cells that fail to divide. HEK293 cells stably expressing Borealin-GFP were exposed to blebbistatin (41 μM). The localization of Borealin and α-tubulin were analyzed by confocal microscopy. (A) Untreated, (B and C) Blebbistatin for 3.8 hrs (D and E) Blebbistatin for 45 min. (F) Borealin dephosphorylation and scheme of the experiment. WT-8 cells were synchronized in mitosis with nocodazole and then released from the block in the presence or absence of blebbistatin. Lysates were collected at the times indicated and analyzed by Western blotting. Western transfers were stripped and reprobed for β-actin as a loading control.

Similar to TD-60 and INCENP, [31], we observed that Borealin was able to migrate to the spindle midzone even when myosin was inhibited and cleavage was blocked with blebbistatin (Fig. 6A–E). Interestingly, Borealin was found to localize to a disorganized, tubulin-containing structure found in cells exposed to blebbistatin. This structure is likely the previously described "telophase disk", a remnant of the spindle midzone found in cytochalasin-treated cells [32](Fig. 6A–E). These experiments indicate that the release of Borealin from microtubules is correlated with the completion of cytokinesis; in cells that divide normally Borealin is no longer associated with microtubules in G1 [18]. To test if blocking cytokinesis affects the phosphorylation of Borealin, we synchronized cells in mitosis with nocodazole. Cells were released from the block in the presence or absence of blebbistatin. In both cases, Borealin was mostly dephosphorylated by 8 hours post release (Fig. 6F). These results indicate that Borealin is dephosphorylated even when cytokinesis is blocked, and further suggests that dephosphorylation is not sufficient to release Borealin from microtubules after mitotic exit.

### Phosphorylation of Borealin during interphase

**Figure 7:**
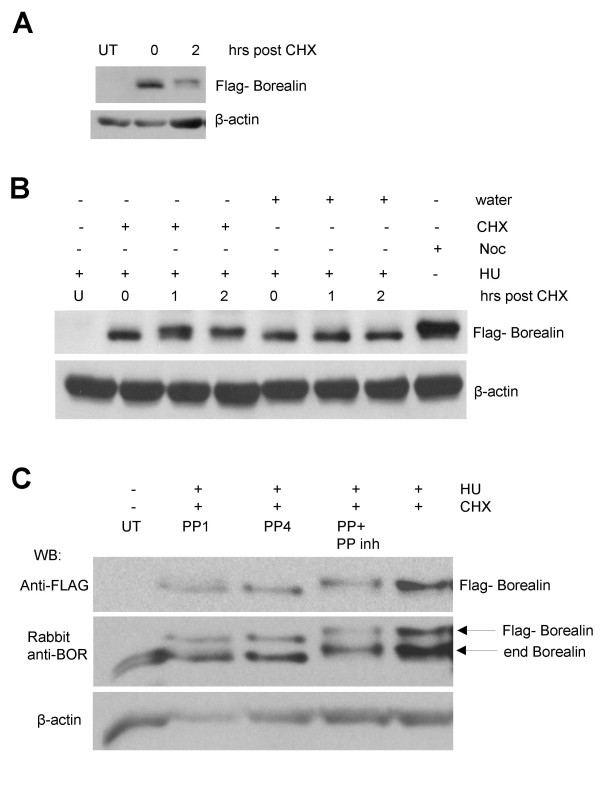
Phosphorylation of Borealin during interphase. Mobility shift of Borealin was analyzed by Western blotting using a 12.6% SDS-PAGE. Western transfers were stripped and reprobed for β-actin as a loading control. UT : Untransfected. (A) Effect of cyclohexamide (CHX) on asynchronous cells. Asynchronously growing WT-8 cells were treated with CHX for the indicated times. (B) Effect of CHX on cells blocked in S phase. WT-8 cells blocked in S phase by treating with hydroxyurea for 16 hours were exposed to CHX or water (vehicle) for 2 hours (in the continuous presence of hydroxyurea). (C) WT-8 cells were either allowed to grow asynchronously or blocked in S phase using hydroxyurea for 16 hours and then treated with CHX for 2 hours. Cell lysates were treated with phosphatase for 1 hr (PP1) and 4 hrs (PP4) *in vitro *and also with phosphatase inhibitor (PP+Inh).

In an experiment aimed at finding the half-life of Borealin protein, asynchronous WT-8 cells were treated with cyclohexamide (CHX) for different time points and the level of Borealin was analyzed. Interestingly, Borealin showed a mobility shift and an abundant slower migrating form after cyclohexamide treatment for 2 hours (Fig. 7A). The short time needed for cyclohexamide to induce the phosphorylation of Borealin suggested that the effect was not secondary to cell cycle synchronization induced by the drug. To confirm this, cells were blocked in S phase with hydroxyurea and then exposed to cyclohexamide for 1 or 2 hours. Even when cell cycle progression was blocked, cyclohexamide induced the phosphorylation of Borealin (Fig. 7B). To confirm that the mobility shift was due to phosphorylation, WT-8 cells blocked in S phase using hydroxyurea were exposed to cyclohexamide for 2 hours and the extracts were then treated *in vitro *with phosphatase for 1 and 4 hours and as a control with phosphatase and phosphatase inhibitor. The disappearance of the slower migrating form upon phosphatase treatment for 4 hours indicated that the shift was due to phosphorylation (Fig. 7C).

### Evidence that the phosphorylation of Borealin is regulated by a labile serine/threonine phosphatase

**Figure 8:**
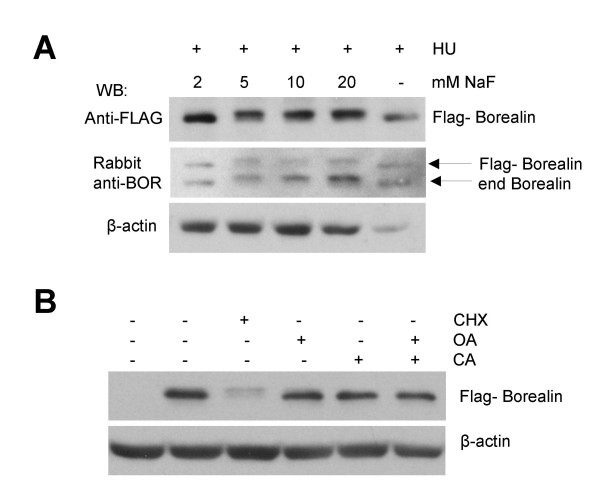
Effect of phosphatase inhibitors on Borealin. The mobility shift of Borealin was determined by Western blotting using 12.6% SDS-polyacrylamide gel. Transfers were stripped and reprobed for β-actin as a loading control. (A) Effect of NaF on the migration of exogenous and endogenous Borealin. WT-8 cells blocked in S phase using hydroxyurea were either left untreated or treated with the indicated doses of NaF for 3.5 hours. (B) Effect of okadaic acid and cyclosporine A on Borealin. Asynchronously growing WT-8 cells were treated with either okadaic acid or cyclosporine A or both for 2 hours. As a control for the shift, cells were treated with CHX for 2 hours.

The effects of cyclohexamide suggest that a kinase that phosphorylates Borealin is present in S phase, and it is possible that it is the same kinase that phosphorylates Borealin during mitosis. One explanation for the effects of cyclohexamide is that a labile phosphatase keeps Borealin dephosphorylated during interphase. Inactivation of the phosphatase during mitosis allows phosphorylated Borealin to accumulate. To test this idea, asynchronously growing WT-8 cells, and those blocked in S phase using hydroxyurea were treated with different concentrations of NaF, a broad-spectrum serine/threonine phosphatase inhibitor for 3.5 hours. Cells treated with 5, 10 and 20 mM NaF showed an increase in the phosphorylated form of Flag-tagged and endogenous Borealin (Fig. 8A). In contrast to NaF, neither okadaic acid nor cyclosporine A had any effect on the migration of Borealin suggesting that Borealin is not dephosphorylated by PP1, PP2A, PP4, PP5 (inhibited by okadaic acid) or PP2B (inhibited by cyclosporine A)(Fig. 8B). Our working hypothesis is that Borealin is constantly phosphorylated during interphase, but is rapidly dephosphorylated by a labile phosphatase.

### Phosphorylated Borealin interacts with INCENP during mitosis

**Figure 9:**
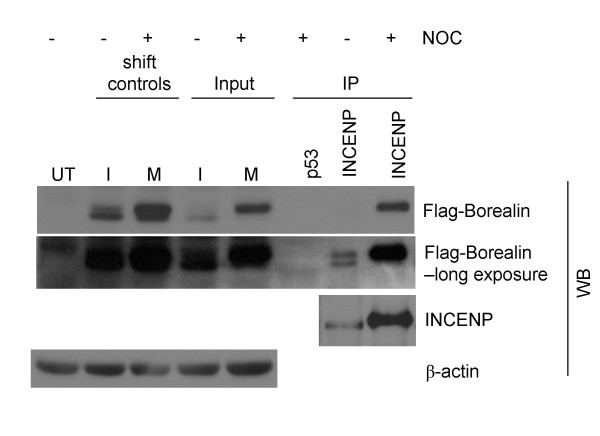
Association of Borealin with endogenous INCENP. Asynchronously growing and mitotic WT-8 lysates were incubated with antibodies to endogenous INCENP or p53 (negative control) for immunoprecipitation and subsequently analyzed by Western blotting (WB) using an antibody to the Flag-tag. Transfers were stripped and probed for INCENP and β-actin as loading control. I: Interphase, M: Mitotic. Shift controls are Hela cells, asynchronously growing or blocked in mitosis with nocodazole and lysed with RIPA buffer. These were included as an internal conformation for the mobility shift and whether electrophoresis was sufficient.

During mitosis, Borealin associates with INCENP-containing complexes[18,20]. To determine which form of Borealin binds to INCENP, immunoprecipitations performed on asynchronous and mitotic WT-8 lysates using anti-INCENP antibodies were analyzed by Western blotting using an antibody to the Flag-tagged Borealin (Fig. 9). Anti-p53 antibody was used as a negative control for immunoprecipitation. In mitotic cells, INCENP-containing complexes almost exclusively contained the phosphorylated form of Borealin (Fig. 9). This was not surprising since most of the Borealin in mitotic cells is phosphorylated. However, even though the asynchronous 'input' lysate contained more of the faster migrating, dephosphorylated form of Borealin, INCENP immunoprecipitates appear to contain equal amounts of both forms of Borealin (Fig. 9). These results indicate that phosphorylation may enhance the interaction of Borealin with the CPC.

## Discussion

The chromosomal passenger complex consisting of INCENP, Survivin, Borealin and Aurora B plays important roles in chromosome segregation at mitosis and cell division once mitosis is complete [1]. The complex shows a dynamic pattern of localization, being found at centromeres during metaphase and being deposited at the spindle midzone during anaphase after sister chromatid separation. There is evidence that targeting to the centromere is in part mediated by Borealin which can bind to DNA [20]. Targeting to the microtubules at the spindle midzone may be mediated by INCENP which has affinity for tubulin [23]. How the localization of the complex is regulated is not completely understood, although the kinesin MKlp-2 is required for re-localization of INCENP and Aurora B from centromeres to the spindle midzone [33]. *borealin *mRNA is cell cycle regulated with maximal expression during G2 and mitosis [18,34]. Also, endogenous Borealin protein levels are increased in cells blocked in mitosis using nocodazole [18,34]. We also observed that ectopic Flag-tagged Borealin driven by a constitute CMV promoter was more abundant in nocodazole-blocked cells (Jacob and Taylor, unpublished observations). Since similar levels of GFP protein are expressed under the control of the CMV promoter in asynchronous and mitotic cells, we predict that the level of Borealin protein in mitosis is under post-transcriptional control. Serine 165 can be phosphorylated *in vitro *by Aurora B Kinase [18], and threonine 106 conforms to a single consensus CDK1 site (S/TP-X-K/R) in Borealin. Mutation of either site did not disrupt the upregulation of ectopic Borealin in mitosis (Fig. 1A and unpublished data), or alter the ability of the Flag-tagged protein to localize to centromeres and the spindle midzone (Fig. 2 and unpublished data). Interestingly, extensive electrophoresis allowed us to detect a phosphorylated form of Borealin that is most abundant during mitosis. This slower migrating, phosphorylated form of Borealin was evident for endogenous Borealin and for exogenous, Flag-tagged Borealin in mitotic cells. In addition, this mitotic form of Borealin was observed when serine 165 or threonine 106 were mutated to alanine (Fig. 1A and unpublished data), and also when Aurora B kinase was inhibited by exposing cells to ZM447439 or VE465. Therefore, Borealin is phosphorylated during mitosis *in vivo *by an unknown kinase, at a site or sites other than or in addition to S165 or T106. It is possible that phosphorylation at T106 or S165 occurs *in vivo *but does not lead to a mobility shift and our results do not exclude this possibility.

We observed that Borealin is dephosphorylated as cells exit mitosis. Interestingly, treating asynchronous or S phase cells with cyclohexamide induced a mobility shift of Borealin as a result of phosphorylation. Our model to explain the effects of cyclohexamide is that the Borealin kinase is active throughout the cell cycle, but is counteracted by a labile phosphatase that is active during interphase. Inactivation of the phosphatase during mitosis would allow accumulation of the mitotic form of Borealin. Consistent with this hypothesis, we observed an increase in the slower migrating, phosphorylated form of Borealin when asynchronous or S phase cells were exposed to NaF, a broad-spectrum phosphatase inhibitor. The shifted form of Borealin induced by cyclohexamide or NaF shows a similar mobility as the form that accumulates during mitosis. Our working hypothesis is that the same phosphorylation events are responsible for these shifted forms. The fact that neither okadaic acid nor cyclosporine A induced the phosphorylation of Borealin during interphase indicates that dephosphorylation is not accomplished by the phosphatases PP1, PP2A, PP2B, PP4 or PP5. There are other more complicated models to explain the effects of cyclohexamide and NaF on Borealin phosphorylation and further study will be required to understand the molecular basis of its cell cycle-dependent phosphorylation.

The mechanisms that control the function of Borealin and the chromosomal passenger complex are not completely understood. For example, as found for INCENP and Aurora B [31] we found that the movement of Borealin from centromeres to the spindle midzone was independent of a functional actomyosin ring. Borealin was found at the telophase disk, a remnant of the spindle midzone that persists hours after cleavage has been aborted [32]. In some cells we could observe decondensed chromatin indicating that the binucleated daughter nuclei had entered G1, with Borealin still attached to the remnants of the spindle midzone (for example see Fig. 6C). The mechanism responsible for this persistent accumulation is not clear. The release of Borealin from the spindle midzone may require additional mechanisms apart from dephosphorylation of Borealin. One possibility is that cell division provides a trigger to remove Borealin (and likely the entire CPC) from the midzone microtubules.

To begin to analyze the functional significance of Borealin phosphorylation, we studied its interaction with another CPC protein, INCENP. Abundant, phosphorylated Borealin was detected in INCENP immunoprecipitates from mitotic cells showing that INCENP interacts with phosphorylated Borealin, the form that predominates during mitosis. Analysis of INCENP immunoprecipitates from asynchronous cells was more informative. Although Borealin was mostly in the dephosphorylated form in the total lysate, INCENP immunoprecipitated equal amounts of both migrating forms of Borealin. This shows that the interphase, fast migrating form of Borealin can form a complex with INCENP, however, preference is given to the phosphorylated form. One interpretation is that the phosphorylation of Borealin during mitosis stabilizes its association with the CPC. This may occur by stabilizing its interaction with Survivin, INCENP, itself, DNA or via a combination of those interactions. Further analysis of the modification of Borealin should help to illuminate how the CPC is assembled.

## Conclusion

Our results indicate that a phosphorylated form of Borealin exists in mitotic cells. Generation of this mitotic phosphorylated form of Borealin does not require Aurora B Kinase activity or the residues S165 or T106 of Borealin. Both the interphase form and the mitotic form of Borealin can form a complex with INCENP, however there is a preference for the mitotic form. A phosphorylated form of Borealin can also be induced in interphase cells by treatment with either cyclohexamide or NaF. Borealin travels to the spindle midzone when actin/myosin-mediated contraction of the cleavage furrow is inhibited, and remains attached to the remnants of the spindle in cells that fail to divide. Borealin is dephosphorylated when cells exit mitosis whether or not they are able to form a functional cleavage furrow.

## Methods

### Cell culture and drug treatment

Cells were maintained in Dulbecco's minimal essential medium (Gibco) with 10% fetal bovine serum and penicillin/streptomycin and in a humidified atmosphere of 10% CO_2 _at 37°C. Chemicals were obtained from Sigma-Aldrich unless otherwise noted. Nocodazole was used at a concentration of 200 ng/ml, LLnL at 50 μM, blebbistatin at 100 μM, hydroxyurea at 2 mM, Cyclohexamide at 25 μg/ml, okadaic acid at 50 nM and cyclosporine A at 100 nM. In experiments where nocodazole was used to synchronize cells, a lower concentration (60 ng/ml for 16 hours) was used to aid in its removal by multiple washes with phosphate buffered saline (PBS). In order to collect mitotic cells in the absence of nocodazole, WT-8 cells stably expressing ectopic Flag-Borealin were plated 1 day before collection. Mitotic cells were collected by vigorous agitation of the plate. Aurora B Kinase inhibitors ZM447439 and VE465 were obtained from Astra Zeneca and Vertex Pharmaceuticals Ltd., respectively.

### Construction of T106A and S165A mutants of Borealin

A cDNA encoding Flag-tagged human Borealin protein was previously created in our lab. This cDNA was subjected to polymerase chain reaction (PCR)-mediated site-directed mutagenesis to create T106A and S165A mutants. For the T106A mutant, the following sets of primers were used. To amplify the first 335-bp fragment, the forward primer (5' AGCAGGCTCCGCGGCCGC 3') and the reverse primer R1 containing the mutation (5' AGATTTCAGGGGAGCCTGAATAG 3') were used. To amplify the second 557-bp fragment, the forward primer F2 containing the mutation (5' CTATTCAGGCTCCCCTGAAATC 3') and the reverse primer R2 (5' TGGGTCGGCGCGCCCACC 3') were used. In the third PCR reaction, the PCR products of the first two reactions were used as templates with F1 and R2 primers to construct the final 892-bp mutated PCR product. The 892-bp fragment between the unique Not1 and Asc1 sites in the wild type cDNA was then replaced by the corresponding PCR product containing the mutation to make the T106A construct.

In the S165A mutant, both S164 and S165 were mutated to alanine. The F1 and R2 primers used for the construction for T106A construct were used for the S165A construct but the following R1 and F2 primers containing the mutation were used: R1 (5' TAGCACGAGCAGCCCTTTTCC 3') and F2 (5' GGAAAAGGGCTGCTCGTGCTA 3'). Competent E. coli were then transformed with the ligation products. After selection on ampicillin plates, plasmids encoding T106A and S165A mutated versions of *borealin *were purified from bacteria and used for transfection. All mutations were confirmed by DNA sequencing.

### Transfections and selection of stable cell lines

For transient transfections, Hela were transfected using either Lipofectamine 2000 (Invitrogen) or Fugene 6 (Roche). For stable cell lines, Hela cells were transfected with a construct encoding Flag-tagged wild type Borealin using Lipofectamine 2000. Selection was initiated by adding 400 μg/ml G418, 48 hours after transfection. Stably transfected cells were obtained two weeks after transfection.

### Western blotting

Cells were lysed in buffer containing 50 mM Tris (pH 8.0), 150 mM NaCl, 1.0% NP-40, 1 mM phenyl methane sulfonyl fluoride (PMSF), 1 mM dithiothreitol (DTT), protease inhibitors (1 μg/ml aprotinin, 2 μg/ml leupeptin, 1 μg/ml pepstatin) and phosphatase inhibitors (1 mM sodium fluoride and 1 mM sodium vanadate) on ice and cell lysates were resolved by sodium dodecyl sulfate-polyacrylamide gel electrophoresis (SDS-PAGE)(12.5% acrylamide at a ratio of 37.5:1 acrylamide: bisacrylamide). In some experiments, in order to better separate phosphorylated and unphosphorylated forms of Borealin, lysates were separated using either 12.6% or 15% acrylamide with a ratio of 29.2:0.8 acrylamide: bisacrylamide [35]. The proteins were subsequently transferred to polyvinylidenefluoride membranes (Millipore, Bedford, MA). Western blots were blocked in PBS containing 0.05%(v/v) Tween and 5%(w/v) non-fat dry milk and antibodies for Western blotting were diluted in the same buffer. Antibodies to Flag-tag were directly conjugated to HRP (Bethyl laboratories) and were used at a dilution of 1:2000. Antibodies to β-actin (NeoMarkers) were diluted to 1:5000, antibodies to GFP (Santa Cruz Biotechnologies) were diluted to 1:500 and antibodies to Cyclin B1 (NeoMarkers) were diluted to 1:300. Rabbit anti-Borealin antibody was used at a dilution of 1:10,000 and anti-INCENP (Upstate) antibody was used at a concentration of 0.5 μg/ml. Goat anti-mouse and goat anti-rabbit secondary antibodies conjugated to HRP (Santa Cruz Biotechnologies) were diluted to 1:2000 and 1:3000, respectively and detected by enhanced chemiluminescence (Dupont, Wilmington, DE)(Clifford, Beljin, Stark & Taylor, 2003).

### Phosphatase treatment

Hela cells transiently transfected with wild-type or T106A Flag-Borealin were left untreated or treated with nocodazole for 14 hrs. Cells were lysed with RIPA buffer (10 mM TRIS pH 7.4, 150 mM NaCl, 1% NP-40, 1% sodium deoxycholate, 0.1% SDS) containing aprotinin, leupeptin, pepstatin A, PMSF and DTT as described above, however sodium fluoride and sodium vanadate were omitted. Cell lysates were left untreated or treated with 1.5 units of calf intestinal phosphatase (Promega) in the reaction buffer provided for 1 or 4 hrs at 37°C. For samples where the added phosphatase was inhibited with phosphatase inhibitors, we also added NaF and NaVO_3_, both at a concentration of 1 mM. Reactions were stopped by adding a Laemlli loading buffer stock to attain the following final buffer composition (2% [w/v] SDS, 50 mM Tris, pH 6.8, 10% [v/v] glycerol and 100 mM DTT).

### Co-Immunoprecipitation

Asynchronously growing or mitotic WT-8 cells (blocked in mitosis using 60 ng/ml nocodazole for 16 hours) were lysed as previously described with some modifications [18]. Cells were incubated in lysis buffer containing 50 mM Tris-HCl (pH 8.0), 400 mM NaCl, 0.5% NP-40, 0.1% deoxycholate, 1 mM PMSF, 1 μg/ml protease inhibitors (aprotinin, leupeptin and pepstatin), 1 mM phosphatase inhibitors (sodium fluoride and sodium vanadate), 30 μg/ml RNase A and 80 U/ml DNase1 for 20 minutes on ice. 4 μg/ml anti-INCENP antibodies and 4 μg/ml anti-p53 antibodies (negative control)(Santa Cruz Biotechnology) coupled to protein-G magnetic beads were added to the cleared lysate and rocked for 5 hours at 4°C. The beads were washed twice with the lysis buffer (without RNase A and DNase1) and once with wash buffer containing 10 mM Tris-HCl (pH 7.4) and 150 mM NaCl. The washed beads were analyzed by SDS-polyacylamide gel electrophoresis and subsequent Western blotting.

### Immunofluorescence and confocal microscopy

For immunofluorescence microscopy, Hela cells grown on coverslips were transiently transfected with constructs encoding Flag-tagged wild type, T106A and S165A mutated versions of *borealin*. 48 hours after transfection, cells were fixed with 100% methanol followed by 100% acetone and blocked with PBS containing 0.1% bovine serum albumin (BSA) overnight at 4°C. Blocked cells were incubated with rabbit anti-Flag (Sigma) and mouse anti-α tubulin (Sigma) primary antibodies for one hour at room temperature. Cells were then incubated with Alexafluor conjugated goat anti-rabbit (Molecular Probes) and fluorescein isothiocyanate-conjugated goat anti-mouse (Sigma) secondary antibodies for 45 minutes at room temperature. Nuclei were stained with 4', 6-Diamidino-2-phenylindole and coverslips were mounted using Vectashield (Vector Laboratories). Standard fluorescent images were captured using a spot camera connected to an Axiophot fluorescence microscope. To obtain confocal images, HEK293 cells stably expressing Borealin-GFP were fixed with 2% formaldehyde in PBS. Cells were permeabilized with 150 mM NaCl, 10 mM Tris (pH 7.7), 0.1% Triton-X-100 (v/v), and 0.1% BSA (w/v). Microtubules were detected with a monoclonal antibody to α-tubulin followed by goat-anti-mouse Alexafluor 568 (Molecular Probes). Borealin was detected by GFP fluorescence and confocal images were captured with an inverted 1 × 70 confocal microscope with Fluoview software (Olympus-Fluoview).

### Production of rabbit anti-Borealin antiserum

To raise polyclonal antisera, a fusion containing glutathione-S-transferase (GST) followed by Borealin at the carboxyl-terminus was created. Full length *borealin *was amplified by PCR using a forward primer containing a Bcl1 site (5-GCGGTGATCACTCCTAGGAAGGGCAGTAGTC-3') and a reverse primer with an EcoR1 site (5'-GAATTCTCATTTGTGGGTCCGTATGCTGCT-3'). The PCR product was digested and ligated into pGEX-3X digested with BamH1 and EcoR1, clones were tested by restriction mapping and confirmed by DNA sequencing. The *gst-borealin *was transformed into Rosetta strain of E. coli (Novagen), which was grown at 37C for 16 h and induced with IPTG for 3 h. Under these conditions, GST-Borealin enters inclusion bodies. To isolate the inclusion bodies, bacterial pellets were resuspended in PBS containing aprotinin, leupeptin, pepstatin A, PMSF and 0.1% lysozyme (w/v). Bacteria were disrupted by sonication and insoluble material collected by centrifugation. The pellet was resuspended in wash buffer (50 mM Tris pH 8.0, 2% (v/v) Triton-X-100, 10 mM EDTA) containing 0.5 M Urea, and insoluble material collected by centrifugation. The pellet was resuspended in wash buffer containing 1 M Urea, and again subject to centrifugation. The pellet was resuspended in wash buffer with 2 M Urea, and insoluble material collected by centrifugation. The pellet, representing washed inclusion bodies was resuspended in PBS and sent to Proteintech Group Inc. for solubilization, injection into rabbits and production of custom antiserum.

## Abbreviations

CPC – Chromosomal Passenger Complex,

INCENP – INner CENtromere Protein,

DMSO – Dimethyl Sulfoxide,

PBS – Phosphate Buffered Saline,

SDS-PAGE – sodium dodecyl sulfate – polyacrylamide gel electrophoresis,

GST – Glutathione-S-Transferase,

GFP – Green Fluorescent Protein,

PMSF – Phenyl Methane Sulfonyl Fluoride,

DTT – Dithiothreitol,

CMV – Cytomegalovirus,

BSA – Bovine Serum Albumin,

LLnL – N-acetyl-L-leucyl-L-leucyl-L-norleucinal,

PCR – Polymerase Chain Reaction.

## Authors' contributions

HK created the Borealin mutants, analyzed them by immunofluorescence, participated in drafting the manuscript and carried out all Western blotting with the following exceptions: DD analyzed GFP driven by CMV, AS analyzed Borealin in response to nocodazole. WRT coordinated the study, collected confocal images, helped to prepare recombinant Borealin, and participated in drafting the manuscript. All authors read and approved the final manuscript and declare that they have no competing interests.

## References

[B1] Vagnarelli P, Earnshaw WC (2004). Chromosomal passengers: the four-dimensional regulation of mitotic events. Chromosoma.

[B2] Hsu JY, Sun ZW, Li X, Reuben M, Tatchell K, Bishop DK, Grushcow JM, Brame CJ, Caldwell JA, Hunt DF (2000). Mitotic phosphorylation of histone H3 is governed by Ipl1/aurora kinase and Glc7/PP1 phosphatase in budding yeast and nematodes. Cell.

[B3] Adams RR, Maiato H, Earnshaw WC, Carmena M (2001). Essential roles of Drosophila inner centromere protein (INCENP) and aurora B in histone H3 phosphorylation, metaphase chromosome alignment, kinetochore disjunction, and chromosome segregation. J Cell Biol.

[B4] Murnion ME, Adams RR, Callister DM, Allis CD, Earnshaw WC, Swedlow JR (2001). Chromatin-associated protein phosphatase 1 regulates aurora-B and histone H3 phosphorylation. J Biol Chem.

[B5] Zeitlin SG, Shelby RD, Sullivan KF (2001). CENP-A is phosphorylated by Aurora B kinase and plays an unexpected role in completion of cytokinesis. J Cell Biol.

[B6] Guse A, Mishima M, Glotzer M (2005). Phosphorylation of ZEN-4/MKLP1 by aurora B regulates completion of cytokinesis. Curr Biol.

[B7] Andrews PD, Ovechkina Y, Morrice N, Wagenbach M, Duncan K, Wordeman L, Swedlow JR (2004). Aurora B regulates MCAK at the mitotic centromere. Dev Cell.

[B8] Lan W, Zhang X, Kline-Smith SL, Rosasco SE, Barrett-Wilt GA, Shabanowitz J, Hunt DF, Walczak CE, Stukenberg PT (2004). Aurora B phosphorylates centromeric MCAK and regulates its localization and microtubule depolymerization activity. Curr Biol.

[B9] Bishop JD, Schumacher JM (2002). Phosphorylation of the carboxyl terminus of inner centromere protein (INCENP) by the Aurora B Kinase stimulates Aurora B kinase activity. J Biol Chem.

[B10] Wheatley SP, Henzing AJ, Dodson H, Khaled W, Earnshaw WC (2004). Aurora-B phosphorylation in vitro identifies a residue of survivin that is essential for its localization and binding to inner centromere protein (INCENP) in vivo. J Biol Chem.

[B11] Minoshima Y, Kawashima T, Hirose K, Tonozuka Y, Kawajiri A, Bao YC, Deng X, Tatsuka M, Narumiya S, May WS (2003). Phosphorylation by aurora B converts MgcRacGAP to a RhoGAP during cytokinesis. Dev Cell.

[B12] Goto H, Yasui Y, Kawajiri A, Nigg EA, Terada Y, Tatsuka M, Nagata K, Inagaki M (2003). Aurora-B regulates the cleavage furrow-specific vimentin phosphorylation in the cytokinetic process. J Biol Chem.

[B13] Kawajiri A, Yasui Y, Goto H, Tatsuka M, Takahashi M, Nagata K, Inagaki M (2003). Functional significance of the specific sites phosphorylated in desmin at cleavage furrow: Aurora-B may phosphorylate and regulate type III intermediate filaments during cytokinesis coordinatedly with Rho-kinase. Mol Biol Cell.

[B14] Murata-Hori M, Fumoto K, Fukuta Y, Iwasaki T, Kikuchi A, Tatsuka M, Hosoya H (2000). Myosin II regulatory light chain as a novel substrate for AIM-1, an aurora/Ipl1p-related kinase from rat. J Biochem (Tokyo).

[B15] Chantalat L, Skoufias DA, Kleman JP, Jung B, Dideberg O, Margolis RL (2000). Crystal structure of human survivin reveals a bow tie-shaped dimer with two unusual alpha-helical extensions. Mol Cell.

[B16] Muchmore SW, Chen J, Jakob C, Zakula D, Matayoshi ED, Wu W, Zhang H, Li F, Ng SC, Altieri DC (2000). Crystal structure and mutagenic analysis of the inhibitor-of-apoptosis protein survivin. Mol Cell.

[B17] Verdecia MA, Huang H, Dutil E, Kaiser DA, Hunter T, Noel JP (2000). Structure of the human anti-apoptotic protein survivin reveals a dimeric arrangement. Nat Struct Biol.

[B18] Gassmann R, Carvalho A, Henzing AJ, Ruchaud S, Hudson DF, Honda R, Nigg EA, Gerloff DL, Earnshaw WC (2004). Borealin: a novel chromosomal passenger required for stability of the bipolar mitotic spindle. J Cell Biol.

[B19] Sampath SC, Ohi R, Leismann O, Salic A, Pozniakovski A, Funabiki H (2004). The chromosomal passenger complex is required for chromatin-induced microtubule stabilization and spindle assembly. Cell.

[B20] Klein UR, Nigg EA, Gruneberg U (2006). Centromere targeting of the chromosomal passenger complex requires a ternary subcomplex of Borealin, Survivin, and the N-terminal domain of INCENP. Mol Biol Cell.

[B21] Lens SM, Rodriguez JA, Vader G, Span SW, Giaccone G, Medema RH (2006). Uncoupling the central spindle-associated function of the chromosomal passenger complex from its role at centromeres. Mol Biol Cell.

[B22] Ainsztein AM, Kandels-Lewis SE, Mackay AM, Earnshaw WC (1998). INCENP centromere and spindle targeting: identification of essential conserved motifs and involvement of heterochromatin protein HP1. J Cell Biol.

[B23] Mackay AM, Eckley DM, Chue C, Earnshaw WC (1993). Molecular analysis of the INCENPs (inner centromere proteins): separate domains are required for association with microtubules during interphase and with the central spindle during anaphase. J Cell Biol.

[B24] Wheatley SP, Kandels-Lewis SE, Adams RR, Ainsztein AM, Earnshaw WC (2001). INCENP binds directly to tubulin and requires dynamic microtubules to target to the cleavage furrow. Exp Cell Res.

[B25] Honda R, Korner R, Nigg EA (2003). Exploring the functional interactions between Aurora B, INCENP, and survivin in mitosis. Mol Biol Cell.

[B26] Goto H, Kiyono T, Tomono Y, Kawajiri A, Urano T, Furukawa K, Nigg EA, Inagaki M (2006). Complex formation of Plk1 and INCENP required for metaphase-anaphase transition. Nat Cell Biol.

[B27] Heinrich MC, Silvey KV, Stone S, Zigler AJ, Griffith DJ, Montalto M, Chai L, Zhi Y, Hoatlin ME (2000). Posttranscriptional cell cycle-dependent regulation of human FANCC expression. Blood.

[B28] Ditchfield C, Johnson VL, Tighe A, Ellston R, Haworth C, Johnson T, Mortlock A, Keen N, Taylor SS (2003). Aurora B couples chromosome alignment with anaphase by targeting BubR1, Mad2, and Cenp-E to kinetochores. J Cell Biol.

[B29] Gadea BB, Ruderman JV (2005). Aurora kinase inhibitor ZM447439 blocks chromosome-induced spindle assembly, the completion of chromosome condensation, and the establishment of the spindle integrity checkpoint in Xenopus egg extracts. Mol Biol Cell.

[B30] Harrington EA, Bebbington D, Moore J, Rasmussen RK, Ajose-Adeogun AO, Nakayama T, Graham JA, Demur C, Hercend T, Diu-Hercend A (2004). VX-680, a potent and selective small-molecule inhibitor of the Aurora kinases, suppresses tumor growth in vivo. Nat Med.

[B31] Martineau-Thuillier S, Andreassen PR, Margolis RL (1998). Colocalization of TD-60 and INCENP throughout G2 and mitosis: evidence for their possible interaction in signalling cytokinesis. Chromosoma.

[B32] Martineau SN, Andreassen PR, Margolis RL (1995). Delay of HeLa cell cleavage into interphase using dihydrocytochalasin B: retention of a postmitotic spindle and telophase disc correlates with synchronous cleavage recovery. J Cell Biol.

[B33] Gruneberg U, Neef R, Honda R, Nigg EA, Barr FA (2004). Relocation of Aurora B from centromeres to the central spindle at the metaphase to anaphase transition requires MKlp2. J Cell Biol.

[B34] Chang JL, Chen TH, Wang CF, Chiang YH, Huang YL, Wong FH, Chou CK, Chen CM (2006). Borealin/Dasra B is a cell cycle-regulated chromosomal passenger protein and its nuclear accumulation is linked to poor prognosis for human gastric cancer. Exp Cell Res.

[B35] Chadee DN, Taylor WR, Hurta RA, Allis CD, Wright JA, Davie JR (1995). Increased phosphorylation of histone H1 in mouse fibroblasts transformed with oncogenes or constitutively active mitogen-activated protein kinase kinase. J Biol Chem.

